# Co-occurring mutations identify prognostic subgroups of microsatellite stable colorectal cancer

**DOI:** 10.1186/s12943-024-02173-x

**Published:** 2024-11-25

**Authors:** Luís Nunes, Jakob Mørkved Stenersen, Kushtrim Kryeziu, Tobias Sjöblom, Bengt Glimelius, Ragnhild A. Lothe, Anita Sveen

**Affiliations:** 1 Department of Molecular Oncology, Institute for Cancer Research, Oslo University Hospital, Oslo, Norway; 2 Institute of Clinical Medicine, Faculty of Medicine, University of Oslo, Oslo, Norway; 3 Department of Immunology, Genetics and Pathology, Science for Life Laboratory, Uppsala University, Uppsala, Sweden

**Keywords:** CRC, Locoregional, Metastatic, MSS, Survival, Prognosis, Co-mutations

## Abstract

**Background:**

Co-occurring mutations in pairs of genes can pinpoint clinically relevant subgroups of cancer. Most colorectal cancers (CRCs) are microsatellite stable (MSS) and have few frequent mutations. Large patient cohorts and broad genomic coverage are needed for comprehensive co-mutation profiling.

**Methods:**

Co-mutations were identified in a population-based Swedish cohort analyzed by whole-genome sequencing (*n*=819 stage I-IV MSS CRCs). Prognostic value was further evaluated in a publicly available dataset of clinically sequenced metastatic CRCs (MSK-IMPACT; *n*=934 MSS). Multivariable Cox proportional hazards analyses with clinicopathological parameters were performed for locoregional (stage I-III) and metastatic (stage IV and recurrent) cancers separately.

**Results:**

Prevalent co-mutations were detected in 23 unique gene pairs, 20 of which included *APC*, *TP53*, *KRAS* and/or *PIK3CA*. Several co-mutations involving *APC* were associated with good overall survival in locoregional CRC, including *APC*-*TCF7L2* (multivariable HR: 0.49, 95% CI 0.27-0.89). This co-mutation was prognostic also in metastatic cancers (multivariable HR: 0.49 and 0.37, 95% CI: 0.24-0.98 and 0.17-0.82 in the Swedish and MSK cohorts, respectively). *APC*-*SOX9* co-mutations were mutually exclusive with *APC*-*TCF7L2*, and the co-mutations combined had stronger prognostic associations than *APC* alone in both metastatic cohorts. *BRAF* p.V600E-*RNF43* co-mutations were associated with poor overall and recurrence-free survival in locoregional CRC (multivariable HR: 4.13 and 3.2, 95% CI: 1.78-9.54 and 1.53-8.04, respectively).

**Conclusions:**

We report a genome-wide evaluation of co-occurring mutations in MSS CRCs, and suggest that co-mutations can improve the prognostic stratification compared to single mutations alone.

**Supplementary Information:**

The online version contains supplementary material available at 10.1186/s12943-024-02173-x.

## Introduction

Most colorectal cancers (CRCs) are microsatellite stable (MSS) and have a moderate mutation rate compared to other solid malignancies. Five to seven driver alterations have been estimated to be sufficient for cancer development [1]. Few CRC-critical mutations have clear prognostic implications, with the notable exception of the poor survival associated with *BRAF* p.V600E, and potentially with mutations of *KRAS*/*NRAS* (RAS) [2]. Mutations with lower prevalence can also have important prognostic impact, as illustrated with the pathogenic *POLE* exonuclease mutations found in approximately 1% of tumors [3]. Over the past 5 years there has been increased focus on subgroups of CRCs defined by co-occurring mutations of gene pairs. In particular, co-mutations of RAS and *TP53* are associated with poor survival after liver resection of metastatic CRCs (mCRCs) [4]. Furthermore, co-mutations of *BRAF* p.V600E and *RNF43* have been associated with improved benefit from BRAF-targeted combination therapy of mCRCs [5]. Most co-mutation studies have focused on genes typically covered by targeted sequencing panels and analyzed in a clinical setting. Broader genomic coverage and larger patient cohorts are needed for more systematic evaluation of co-occurring mutations and their prognostic implications.

## Results and discussion

### Patient cohorts and prognostic gene mutations

**Fig. 1:**
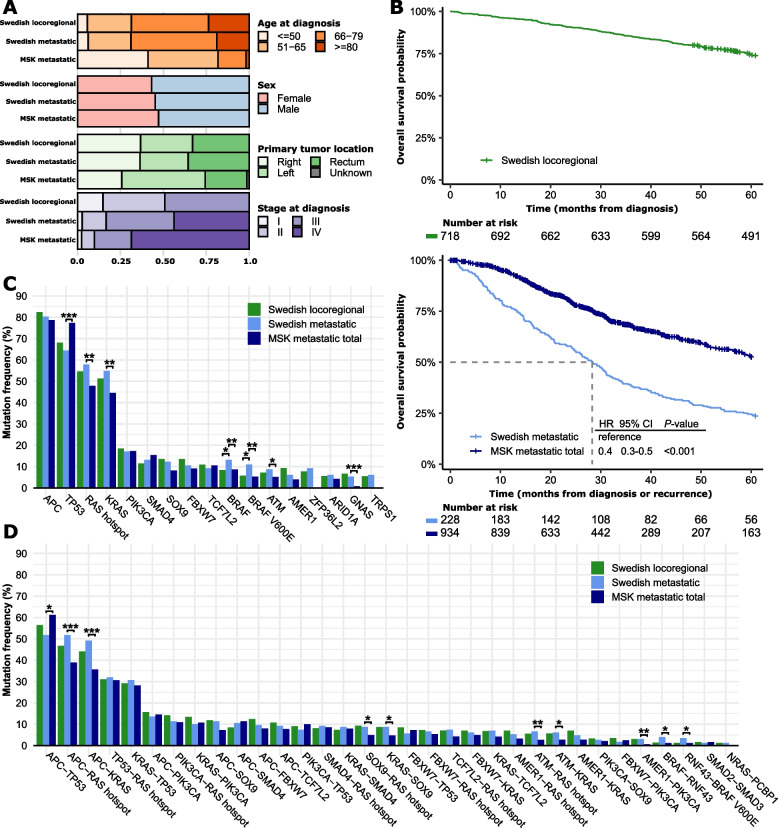
Comparison of clinicopathological parameters and mutations among cohorts.** A** Clinicopathological characteristics of the Swedish locoregional (*n* = 719; 1 patient with missing survival information), Swedish metastatic (*n* = 228; 128 patients with metachronous metastases overlap with the locoregional cohort), and MSK (all patients, *n* = 934) cohorts. **B** Kaplan–Meier plots of overall survival in each cohort. **C-D** Bar plots of the frequency of single (**C**) and co-occurring (**D**) gene mutations in each cohort. Fisher exact analyses were performed between the cohorts and statistically significant differences are indicated by asterisks (***p < 0.001, **p < 0.01, and *p < 0.05)

Co-mutation discovery focusing on nonsynonymous single nucleotide variants (SNVs) and small insertions and deletions (indels) was performed in a Swedish population-based series of stage I-IV MSS CRCs analyzed by whole-genome sequencing (*n* = 819) [6]. Survival analyses were performed separately for locoregional (*n* = 719 stages I-III) and metastatic cancers (*n* = 228; Fig. 1A and Supplementary Table 1) using overall survival (OS) as the primary endpoint, and recurrence-free survival (RFS) as secondary endpoint for locoregional cancers. The metastatic cohort included patients diagnosed with synchronous metastases (*n* = 100 stage IV, 44%) and recurrent cases from the locoregional cohort (*n* = 128). Additional prognostic analyses were performed in a publicly available single-hospital series of MSS mCRCs sequenced with the MSK-IMPACT gene panel (*n* = 934, 69% diagnosed with stage IV; Fig. 1A and Supplementary Table 1) [7]. The MSK metastatic cohort had younger patient age, less frequent right-sided primary tumors, and better OS than the Swedish metastatic cohort (Fig. 1A-B and Supplementary Table 1). Clinicopathological parameters with prognostic associations were included with the mutations in multivariable survival models (Supplementary Fig. 1). Metastasectomy status was a strong prognostic factor in both metastatic cohorts (Supplementary Fig. 1) and was included for stratified analyses of the MSK cohort (Supplementary Fig. 2A-B and Supplementary Table 2). The Swedish metastatic cohort was not sufficiently sized for stratified analyses, but there was no difference in the frequency of metastasectomy between the two cohorts (Supplementary Table 1).

**Fig. 2:**
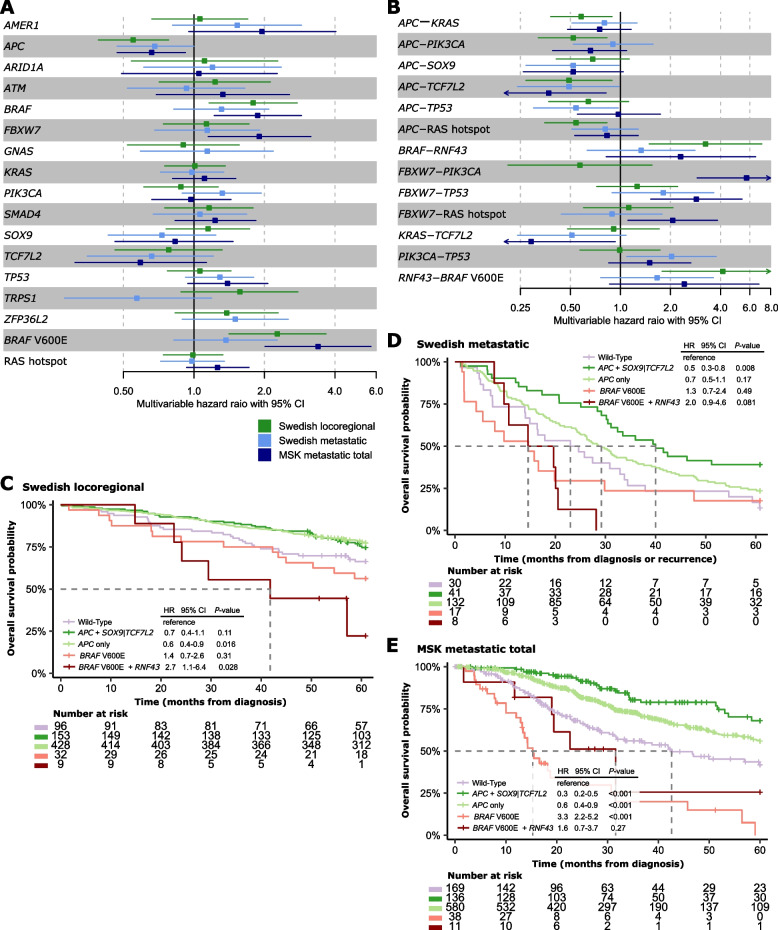
Survival analyses of single mutations and co-occurring mutations in each cohort. **A-B** Forest plots for multivariable Cox proportional hazards models of overall survival according to each of the most frequent (**A**) single mutations and (**B**) co-mutations in each cohort. The reference group is cancers without mutations in the respective genes. Only co-mutations with significant prognostic associations (*p* < 0.05) in at least one cohort are shown. **C**-**E** Kaplan–Meier plots of overall survival according to selected co-mutations in each cohort. The wild-type group represents patients without mutations in any of the genes considered, and was used as reference group in statistical comparisons. Hazard ratios are from Cox proportional hazard analyses, and *p*-values from Wald’s tests. One patient in the Swedish locoregional cohort had missing survival information

Among the most frequently mutated genes in the full Swedish cohort (*n* = 15 genes with SNVs or indels in ≥ 5% of tumors), only *BRAF* had a different mutation frequency between the locoregional and metastatic/recurrent cancers (*BRAF*: 8% vs 13%, *p* = 0.04; *BRAF* p.V600E: 6% vs 11%, *p* = 0.01; Fig. 1C). The Swedish metastatic cohort had more frequent *ATM*, *BRAF*, *GNAS*, and *KRAS* (including RAS hotspot) mutations and less frequent *TP53* mutations than the MSK cohort. *APC* mutations were associated with good OS and *BRAF* p.V600E mutations with a poor OS among both locoregional and metastatic cancers (Fig. 2A and Supplementary Table 3). Results were similar for RFS as endpoint in the locoregional cohort (Supplementary Table 3). In a stratified analysis of the MSK cohort, several genes had prognostic associations in either the metastasectomy group (poor prognosis with *FBXW7* mutations) or the no-metastasectomy group (poor prognosis with *ATM* and *TP53* mutations and good prognosis with *SOX9* mutations; Supplementary Fig. 2D). Among these, only *FBXW7* mutations had prognostic associations in the total MSK cohort, and none were prognostic in the Swedish metastatic cohort.

The frequency of copy number amplifications (5 or more additional copies) and homozygous deletions (complete loss) of each of the 15 genes was lower than 6% in each cohort (Supplementary Fig. 3A-B). Incorporation of these copy number alterations had little impact on the prognostic associations of each gene, with the exception that mutation or deletion of *SMAD4* was associated with a poor OS in the MSK cohort (Supplementary Table 4).

### Co-mutations and prognostic associations

Co-mutations were defined as gene pairs with co-occurring SNVs or indels in at least 10% of tumors with mutation of each gene. A total of 33 co-mutations with prevalence above 5% were identified in the full Swedish cohort, of which 23 involved unique gene pairs (not counting hotspot mutations of RAS or *BRAF* p.V600E separately; Fig. 1D). The frequency of co-mutations corresponded largely with the frequency of the individually mutated genes, and only 3 co-mutations did not involve *APC*, *TP53*, *KRAS* and/or *PIK3CA* (*BRAF*-*RNF43*, *NRAS*-*PCBP1*, *SMAD2*-*SMAD3*). The frequency of co-mutations was similar between locoregional and metastatic cancers in the Swedish cohort, as well as between the Swedish metastatic and MSK metastatic cohorts, with the exception that the MSK cohort had more frequent *APC*-*TP53* co-mutations and less frequent co-mutations of RAS hotspots with *APC*, *SOX9* and *ATM*, as well as *BRAF* p.V600E-*RNF43* and *AMER1*-*PIK3CA*.

Co-mutations previously reported to be prognostic in CRC were evaluated specifically, but neither RAS-*TP53* [4], *APC*-*PIK3CA* [8] nor *KRAS*-*PIK3CA* [9] were associated with poor survival in any of the cohorts (Supplementary Table 5). This inconsistency might be related to more heterogeneous patient populations in our study, considering that the previous studies included patients with resectable liver metastases only [4, 8]. Furthermore, *RNF43* mutations have been proposed to be associated with improved survival after BRAF-targeted combination therapy of *BRAF* p.V600E mCRCs [5]. In our study, *BRAF* p.V600E-*RNF43* co-mutations were associated with poor OS and RFS among patients with locoregional cancer (multivariable HR: 4.13 and 3.2, 95% CI: 1.78–9.54 and 1.53–8.04, respectively; Fig. 2B), and had prognostic value also in univariable models of OS in both metastatic cohorts (Supplementary Table 5). However, there was no added prognostic effect of the co-mutation compared to *BRAF* p.V600E alone (Supplementary Fig. 4), consistent with the lack of a prognostic value of *RNF43* in *BRAF* p.V600E mCRCs not receiving targeted treatment [5]. In the Swedish locoregional cohort, patients with the co-mutation had a numerically shorter median OS and RFS than patients with *BRAF* p.V600E alone (Fig. 2C and Supplementary Fig. 4A-B), suggesting a higher prognostic effect of the co-mutation in locoregional compared to metastatic MSS CRCs. However, the survival difference was not statistically significant, and the small sample size of the mutation subgroups precluded conclusions of added prognostic value. The lack of a locoregional validation cohort is a limitation of this study.

Consistent with the prognostic value of *APC* mutations alone, co-mutations of *APC* with *KRAS*, *PIK3CA*, *TCF7L2* and RAS hotspots were associated with good OS among patients with locoregional cancer (Fig. 2B). The prognostic association was consistent among mCRCs for *APC*-*TCF7L2* (multivariable HR: 0.49 and 0.37, 95% CI: 0.24–0.98 and 0.17–0.82 in the Swedish and MSK cohorts, respectively; Fig. 2B), but limited to patients not treated by metastasectomy in the MSK cohort (Supplementary Fig. 2E). Similarly, *APC*-*SOX9* co-mutations were also associated with a good prognosis among mCRCs, and limited to patients not treated by metastasectomy in the MSK cohort (multivariable HR: 0.52 and 0.52, 95% CI: 0.27–0.98 and 0.26–1.04 in the Swedish and total MSK cohorts, respectively; Supplementary Fig. 2E). There was no difference in the frequency of *APC*-*TCF7L2* co-mutations according to metastasectomy status in the MSK cohort, but *APC*-*SOX9* co-mutations were less frequent in the cancers not treated by metastasectomy (Supplementary Fig. 2C).

Several co-mutations involving *FBXW7* had poor-prognostic associations among mCRCs in the MSK cohort, but were either too rare for analysis or not prognostic in the Swedish metastatic cohort (Supplementary Table 5). Co-mutations of *FBXW7* and *PIK3CA* were prognostic both among patients treated and not treated by metastasectomy, while co-mutations of *FBXW7* and *TP53* or RAS hotspots had significant prognostic associations only in the no-metastasectomy and metastasectomy cohorts, respectively (Supplementary Fig. 2E). Incorporation of gene amplifications and homozygous deletions (in addition to SNVs and indels) had little impact on the prognostic associations of co-mutated gene pairs (Supplementary Table 6). However, co-occurrence of mutation or deletion of *SMAD4* with RAS hotspot mutations was associated with a poor survival in the MSK metastasectomy cohort (multivariable HR 4.0, 95% CI 1.7–9.4; Supplementary Fig. 3C-D).

### Co-mutations can enhance the prognostic effect

Co-mutations involving *APC* had prognostic value beyond the effect of the individual genes in the metastatic cohorts (Fig. 2C-E). Co-mutations of *APC* with *TCF7L2* or *SOX9* were mutually exclusive, and *APC*-*TCF7L2*/*SOX9* mutations combined were found in 18% and 15% of mCRCs in the Swedish and MSK cohorts, respectively (*p* = 0.22 by Fisher exact test). These patients had a better OS than patients with *APC* mutations alone (Swedish metastatic cohort HR: 0.65, 95% CI: 0.42–1, *p* = 0.05, and MSK cohort HR: 0.60, 95% CI: 0.39–0.91, *p* = 0.016). There were no differences in clinicopathological features between patients with co-mutations and *APC* mutations alone, but co-mutated cancers had a lower frequency of *TP53* mutations (Supplementary Tables 7–9). The consistent prognostic value of the co-mutation in two metastatic cohorts with substantial differences in clinicopathological features support robustness. Both SOX9 and TCF7L2 are transcription factors involved in the WNT signaling pathway. This pathway is activated in most MSS CRCs due to inactivating mutations of *APC* and failure to sequester the transcriptional co-activator β-catenin. Active β-catenin binds to TCF7L2 and activates the transcription of WNT target genes, including *SOX9* [10]. Overexpression of SOX9 inhibits the β-catenin-TCF7L2 complex to reduce WNT pathway activity [11]. *SOX9* expression was higher in tumors with *APC*-*SOX9* co-mutations in the Swedish cohort (Supplementary Fig. 5), and the positive prognostic effect of *APC*-*TCF7L2*/*SOX9* co-mutations suggests that disruption of the balance between WNT pathway activation (due to loss of APC activity) and its modulation by TCF7L2 or SOX9 might partially reduce the oncogenic effects, leading to less aggressive cancers.

Neither *APC* mutations nor any of the co-mutations involving *APC* were prognostic in the MSK metastasectomy cohort separately (Supplementary Fig. 6A-B). However, the high OS rate and distribution of clinicopathological features suggest that this patient cohort is not population-representative (Supplementary Fig. 2A and Supplementary Table 2), and prognostic data should be interpreted with care. Nonetheless, co-mutations of *FBXW7* with *PIK3CA* and/or RAS hotspots identified a small subgroup of patients in this cohort (4%) who had a poor survival, also compared to patients with *PIK3CA* and/or RAS hotspot mutations alone, supporting the potential of co-mutations to improve the prognostic stratification (Supplementary Fig. 6C-D). There were no differences in clinicopathological features or other mutations between patients with co-mutations and the individually mutated genes (Supplementary Table 10). It has also previously been reported that *FBXW7* mutations predominantly occur alongside with *KRAS* mutations in advanced CRC [12], and both *FBXW7* and RAS mutations have been associated with worse survival after liver resection for mCRC [13]. *FBXW7* mutations can cause increased signaling in the EGFR pathway, similarly to RAS mutations [14], and the poor-prognostic effect of the co-mutations might therefore reflect enhanced oncogenic activity of the EGFR pathway.

## Conclusion

We report genome-wide profiling of co-occurring mutations in MSS CRCs, and suggest that co-mutations can improve the prognostic stratification compared to single mutations alone. In particular, co-mutations of *APC* with *TCF7L2* or *SOX9* may identify a subgroup of metastatic cancers with favorable prognosis.

## Supplementary Information

Supplementary Material 1.

Supplementary Material 2.

## Data Availability

No datasets were generated or analysed during the current study.

## Abbreviations

CI: Confidence interval
CRC: Colorectal cancer
HR: Hazard ratio
mCRC: Metastatic colorectal cancer
MSS: Microsatellite stable
OS: Overall survival
RFS: Recurrence free survival
