# Supplementary material for: Co-occurring mutations identify prognostic subgroups of microsatellite stable colorectal cancer
Source: Mol Cancer. 2024 Nov 25;23:264. doi: 10.1186/s12943-024-02173-x (PMC11587607; doi:10.1186/s12943-024-02173-x)
Supplement: Supplementary file 1 — Supplementary Material 1. [file 12943_2024_2173_MOESM1_ESM.docx]

**Co-occurring mutations identify prognostic subgroups of microsatellite stable colorectal cancer**

Luís Nunes^1^, Jakob Mørkved Stenersen^1^, Kushtrim Kryeziu^1^, Tobias Sjöblom^3^, Bengt Glimelius^3^, Ragnhild A. Lothe^1,2^, Anita Sveen^1,2^

^1^ Department of Molecular Oncology, Institute for Cancer Research, Oslo University Hospital

^2^ Institute of Clinical Medicine, Faculty of Medicine, University of Oslo, Oslo, Norway

^3^ Department of Immunology, Genetics and Pathology, Science for Life Laboratory, Uppsala University, Uppsala, Sweden

**Supplementary Information Guide**

**Supplementary Figures**

Supplementary Figure 1…………………………………………………………………...2

Supplementary Figure 2…………………………………………………………………...3

Supplementary Figure 3…………………………………………………………………...4

Supplementary Figure 4…………………………………………………………………...5

Supplementary Figure 5…………………………………………………………………...6

Supplementary Figure 6…………………………………………………………………...7

**Supplementary Methods**

Clinical data collection of the Swedish cohort……………………………………………8

Clinical data collection of the MSK cohort……………………………………………….8

Genomic data collection and analyses…………………...………………………………..9

Statistical analyses…………………….…………………………………………………..9

**
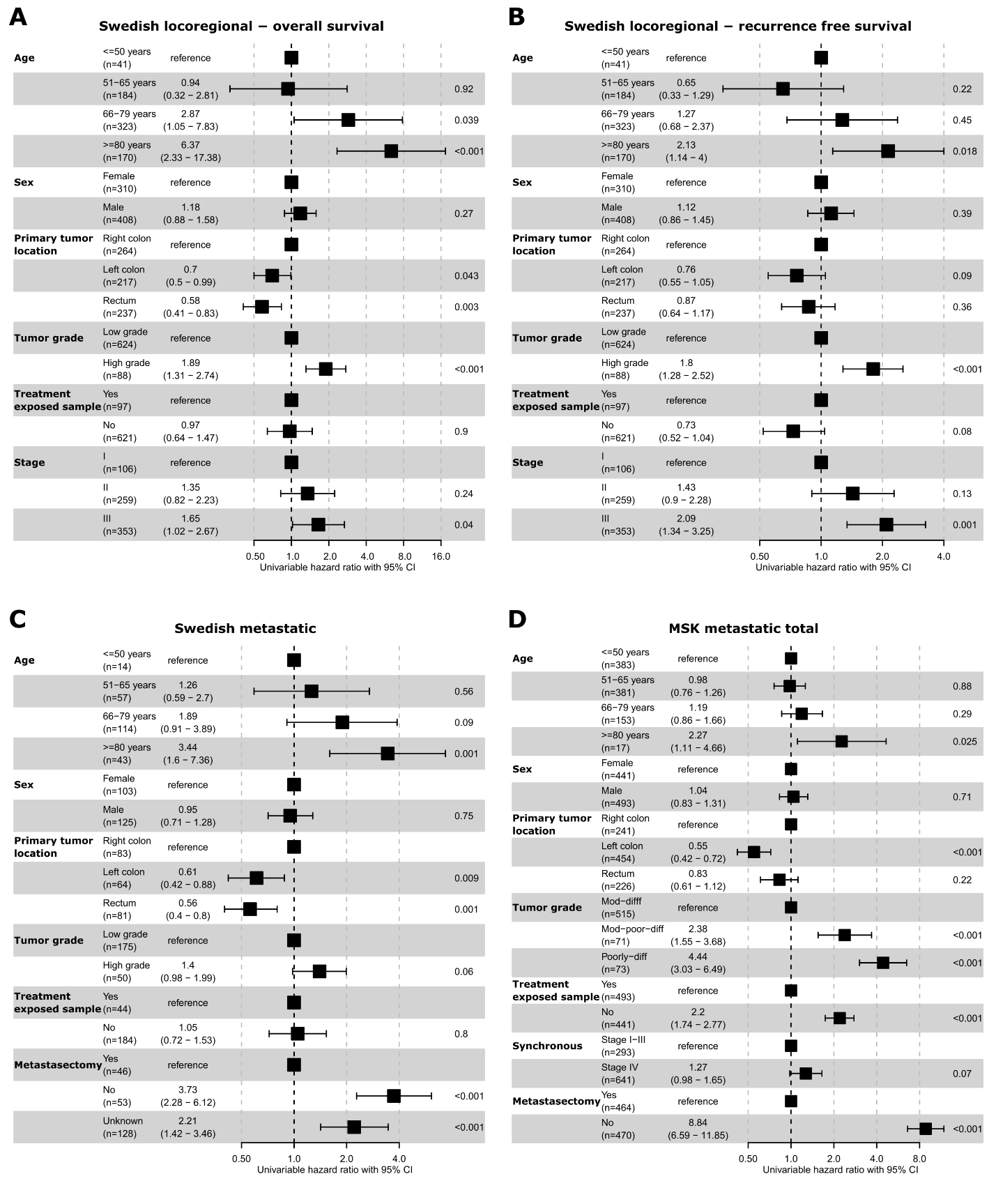
**

**Supplementary Figure 1. Clinical characteristics and survival associations in each cohort. (A-D)** Forest plots for univariable Cox proportional hazards models with clinicopathological characteristics included in the survival analyses for the Swedish locoregional **(A)** overall survival and **(B)** recurrence free survival, **(C)** Swedish metastatic, and **(D)** MSK metastatic total cohorts.

**
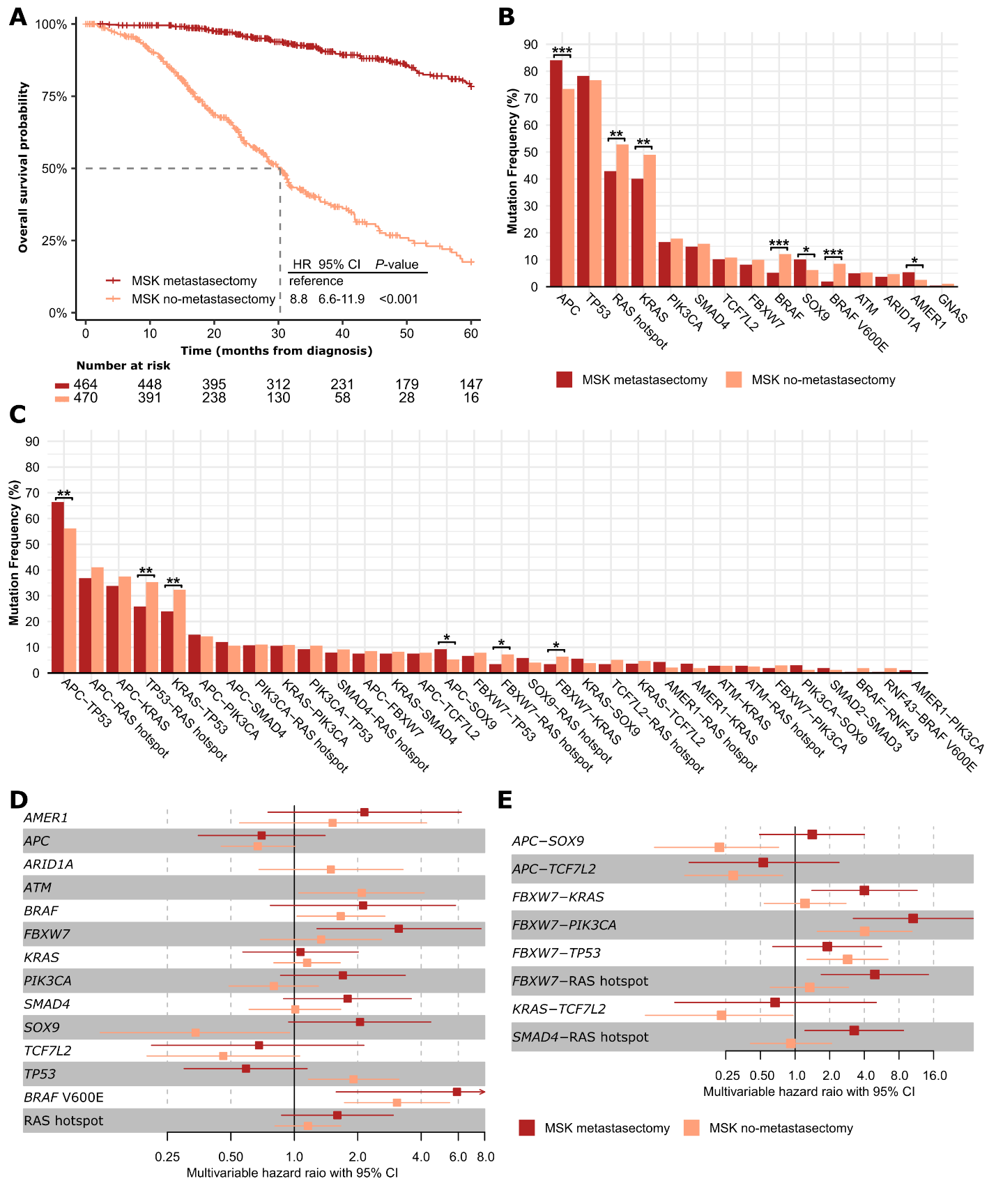
**

**Supplementary Figure 2. Comparison of clinicopathological parameters, mutations and survival among MSK cohort stratified by metastasectomy status.** **(A)** Kaplan-Meier plot of overall survival. **(B-C)** Bar plots of the frequency of single **(C)** and co-occurring **(D)** gene mutations. Fisher exact analyses were performed between the cohorts and significant differences are indicated by asterisks (*** *p* <0.001, ** *p* < 0.01, and * *p* <0.05). **(D-E)** Forest plots for multivariable Cox proportional hazards models of each of the most frequent **(D)** single mutations and **(E)** co-mutations. Only co-mutations with significant prognostic associations (*p* <0.05) in at least one group are shown.

**
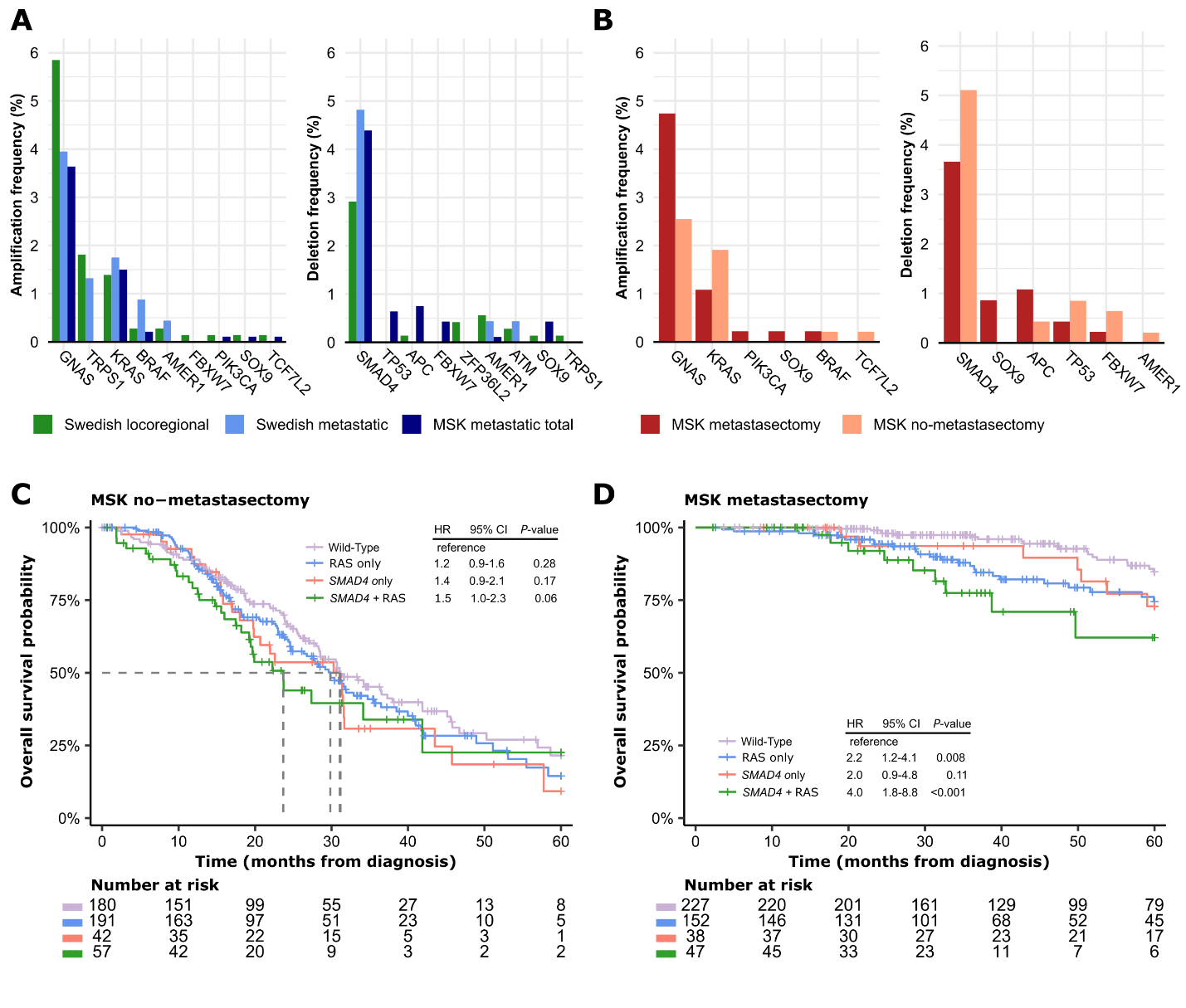
**

**Supplementary Figure 3. Copy number alterations among the Swedish and MSK cohorts, and survival analyses of selected alterations.** Bar plots of the frequency of copy number amplifications and deletions for the **(A)** Swedish (locoregional and metastatic) and MSK cohorts, and **(B)** by metastasectomy status for the MSK cohort. Kaplan-Meier plots of overall survival according to mutations of *SMAD4* (mutations or deletions) and/or RAS (hotspot mutations) in the MSK **(C)** no-metastasectomy and **(D)** metastasectomy cohort. Patients without any of the considered mutations were allocated in the wild-type group.

**
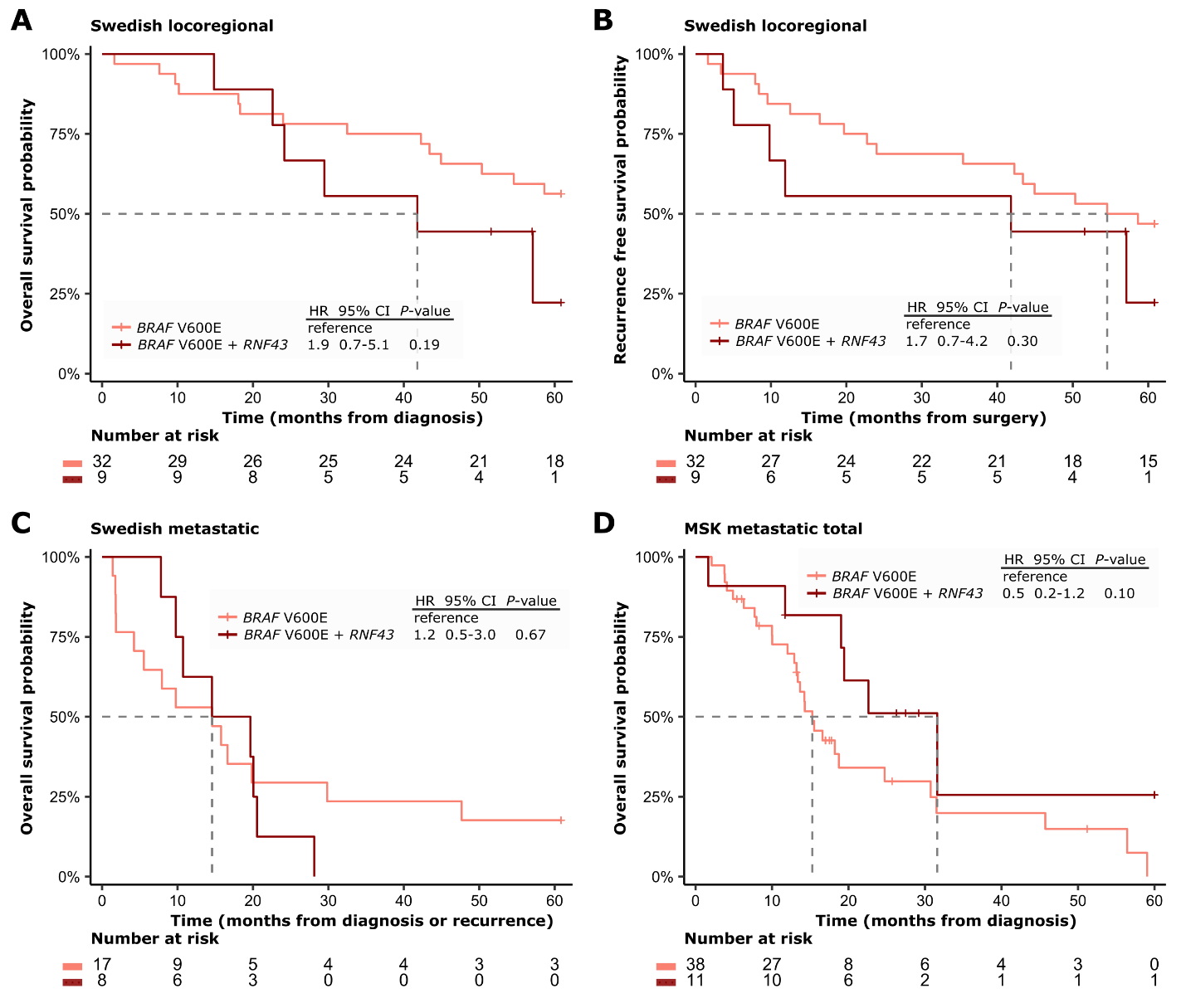
**

**Supplementary Figure 4. Survival analyses of *BRAF* and *RNF43* mutations.** Kaplan-Meier plots of **(A, C-D)** overall and **(B)** recurrence free survival according to *BRAF* p.V600E and *RNF43* mutations in the **(A-B)** Swedish locoregional, **(C)** Swedish metastatic, and **(D)** MSK metastatic total cohorts.

**
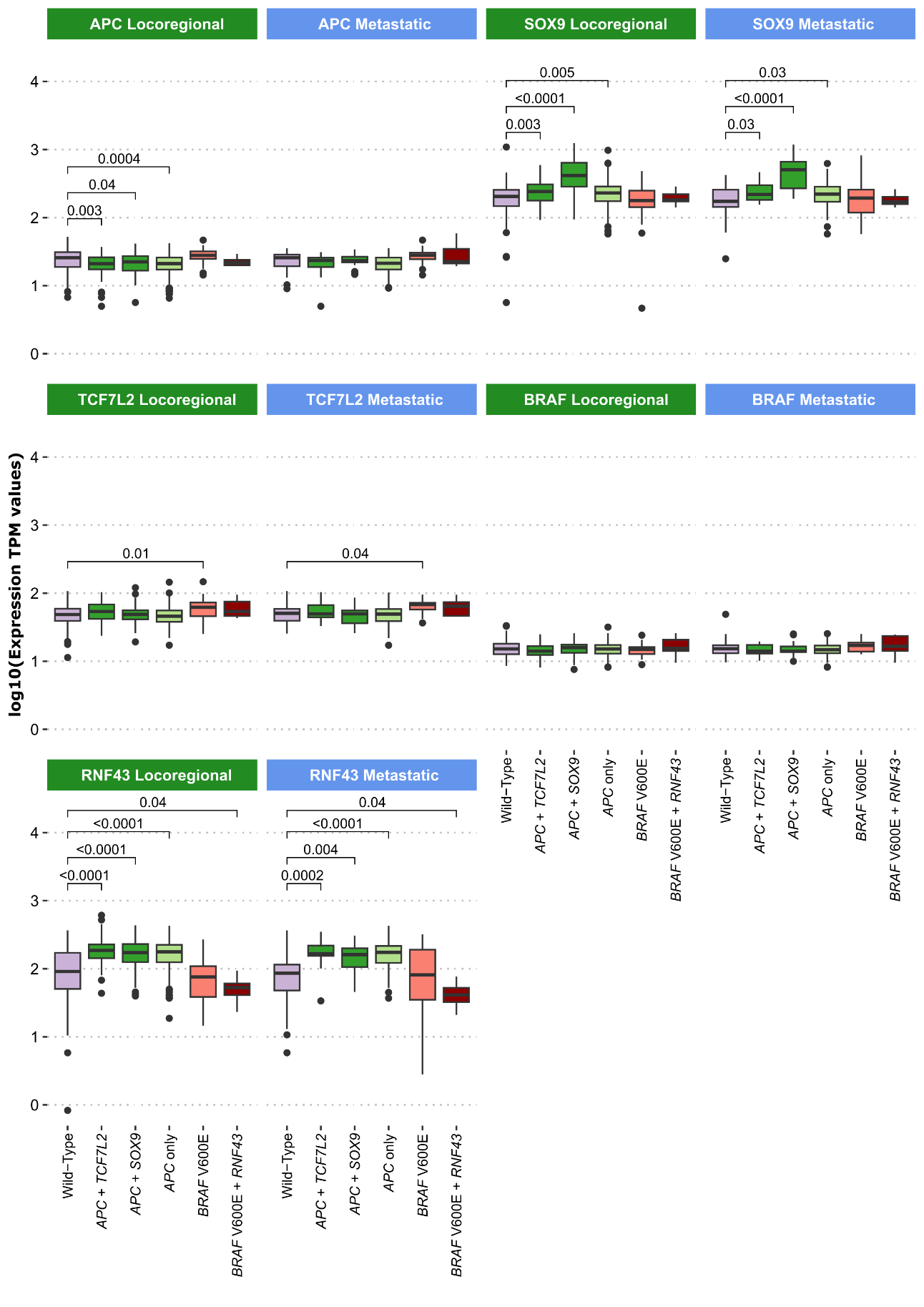
**

**Supplementary Figure 5. Expression level of selected genes according to mutation status in the Swedish locoregional and metastatic cohorts.** P-values were calculated with Wilcoxon test using the wild-type group as the reference, and adjusted with FDR. The boxes represent the interquartile ranges, center lines the median, the whiskers extend 1.5 times the top and bottom of the boxes, and the black dots the outliers.

**
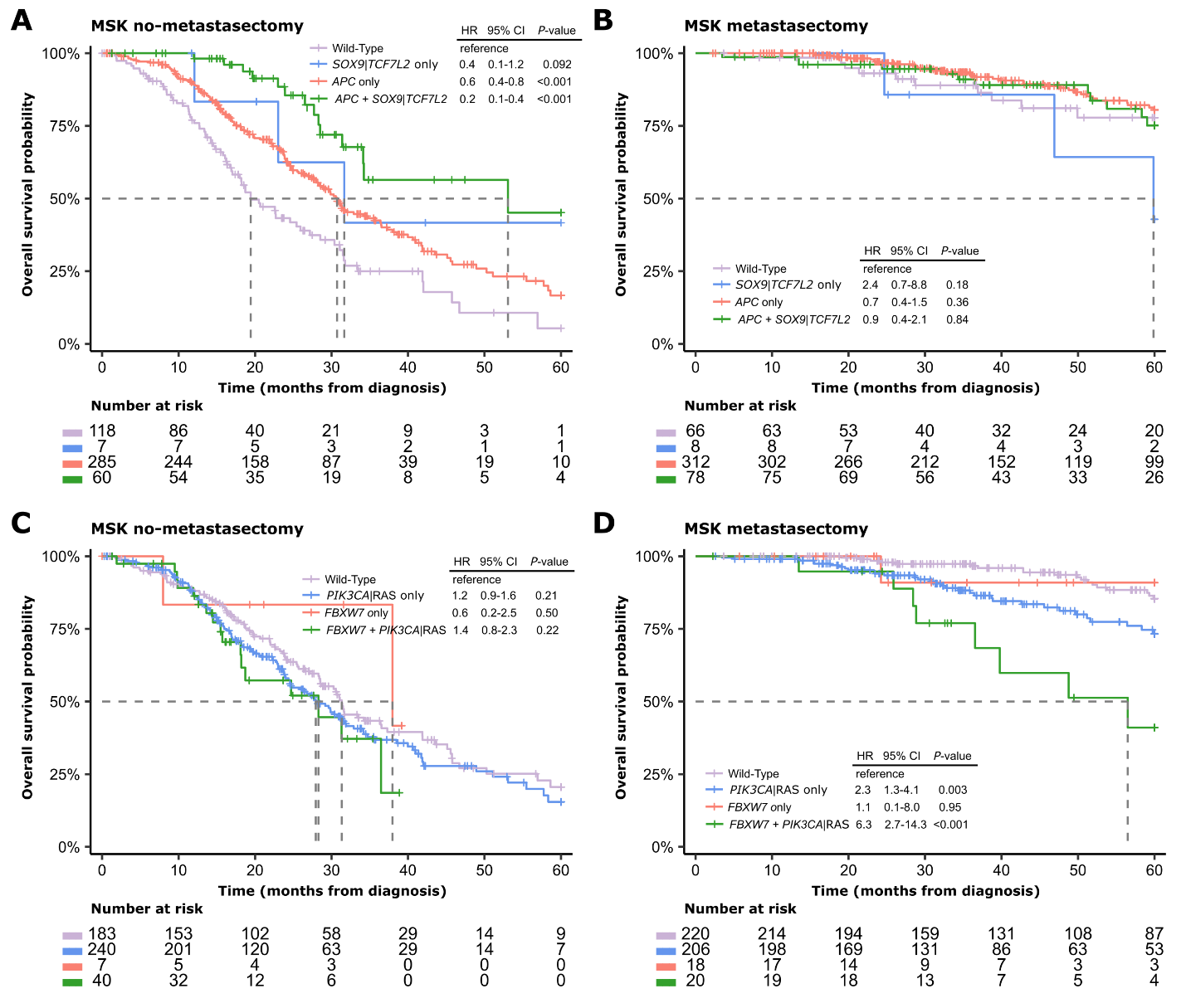
**

**Supplementary Figure 6. Survival analyses of selected mutations.** Kaplan-Meier plots of overall survival according to *APC-SOX9/TCF7L2* co-mutation and respective single mutations for the MSK cohort by **(A)** no-metastasectomy and **(B)** metastasectomy status. Kaplan-Meier plots of overall survival according to *FBXW7-PIK3CA/*RAS co-mutation and respective single mutations for the MSK cohort by **(C)** no-metastasectomy and **(D)** metastasectomy status. Patients without any of the considered mutations were allocated in the wild-type group.

**Supplementary Methods**

**Clinical data collection of the Swedish cohort**

Clinical and genomic data were retrieved from the supplementary data of the associated study[1], and from the accession number PRJEB61514 in the EVA and E-MTAB-12862 in the ArrayExpress public repositories. From a total of 1,063 patients included in the study, 819 cancers were reported as microsatellite stable (MSS) and non-hypermutated and were selected for analysis. Clinicopathological parameters included age at diagnosis, sex, primary tumor location, tumor grade, treatment exposed sequenced sample (indicating treatment exposure prior to sampling), stage, metastasectomy status (available for cancers diagnosed as stage IV only, not for recurrences), and survival outcomes. Overall survival was estimated from time of diagnosis in the locoregional cohort (stages I-III), and from time of diagnosis of metastasis in the metastatic cohort, including synchronous metastasis (stage IV) and earliest local or distant recurrence from locoregional disease. Recurrence free survival was estimated from time of surgery to earliest local or distant recurrence or death in the locoregional cohort.

**Clinical data collection of the MSK cohort**

Clinical and genomic data were obtained from cBioPortal for the publicly available study “Metastatic Colorectal Cancer (MSK, Cancer Cell 2018)”[2]. Of the 1,134 patients included in the study, 934 metastatic MSS cases were selected, characterized by a tumor mutation burden ≤25 mutations per megabase, an MSIsensor score <10, and exclusion of “early stage primary” tumors. Clinicopathological data included age at diagnosis, sex, primary tumor location, primary tumor grade, chemo exposed sequenced sample, stage, metastasectomy status and overall survival outcome. Overall survival was estimated from the diagnosis of metastatic disease. Metastasectomy involved resection of liver metastases (n=364 patients, 74%), lung metastases (n=37, 8%), liver and lung metastases (n=6), liver metastases and other tissue (n=14), and other tissues (n=64, 14%).

**Genomic data collection and analyses**

Genomic data for the Swedish cohort included somatic single nucleotide variants (SNVs) and small insertions and deletions (indels) in 96 genes identified as significantly mutated by dNdScv (refer to Figure 1 from the original study)[1]. Single-gene analyses were conducted for genes with a mutational frequency >5% among the MSS cases. Hotspot mutations in *BRAF* (p.V600E) and RAS (*KRAS* and *NRAS* codons G12/G13/Q61/K117/A146) were analyzed separately. Co-mutation analyses were performed for gene pairs with a co-occurring mutation in >10% of samples with each mutation, and a co-mutational frequency >5% in total. For the genes selected for further analyses, information on copy number amplifications (total copy number, tcn ≥ 5, corresponding to 5 additional copies) and homozygous deletions (tcn and lcn = 0) that passed the filter defined by FACETS were included. For the MSK cohort, genomic data included somatic SNVs, small indels, amplifications and deletions for the genes identified from analyses of the Swedish cohort. The genes *PCBP1*, *TRPS1* and *ZFP36L2* were not included in the MSK gene panel, and *TCF7L2* was only sequenced in some cases. RNA sequencing data was available for the Swedish cohort, and TPM values were used for expression analyses of selected genes.

**Statistical analyses**

All statistical analyses were performed using R version 4.3.2[3]. Cox proportional hazards models and Kaplan-Meier survival estimates were generated using the R package *survival* version 3.7-0, with *p*-values from Wald’s tests. Patients were censored at 5 years of follow-up. Multivariable Cox models included clinicopathological variables with prognostic associations in univariable analyses. For the Swedish locoregional cohort, this included age, sex, primary tumor location, tumor grade, treatment exposure, and stage. The same covariables were included for the Swedish metastatic cohort, except exchange of stage by metastasectomy status (information available for the patients diagnosed with synchronous metastases). The multivariable Cox model for the MSK cohort included age, sex, primary tumor location, tumor grade, pre-treatment status, synchronous vs. metachronous metastasis, and metastasectomy status. The proportional hazard assumption was tested and found to be valid. To account for multiple comparisons in univariable analyses, p-values were adjusted using the false discovery rate (FDR) method. Forest plots were visualized with the R packages *forestplot* version 3.1.3 and *survminer* version 0.4.9. Kaplan-Meier curves were created using the *ggsurvfit* package version 1.1.0. Fisher’s exact test was applied for group comparisons of clinicopathological parameters and mutations. Expression values were compared with Wilcoxon test and p-values were adjusted with the FDR method. All statistical tests were performed with two-sided p-value <0.05 as a threshold of significance.

**Supplementary methods references**

1. Nunes L, Li F, Wu M, Luo T, Hammarström K, Torell E, et al. Prognostic genome and transcriptome signatures in colorectal cancers. Nature. 2024;633:137–46.

2. Yaeger R, Chatila WK, Lipsyc MD, Hechtman JF, Cercek A, Sanchez-Vega F, et al. Clinical Sequencing Defines the Genomic Landscape of Metastatic Colorectal Cancer. Cancer Cell. 2018;33:125-136.e3.

3. R Core Team. R: A Language and Environment for Statistical Computing [Internet]. Vienna, Austria: R Foundation for Statistical Computing; 2021. Available from: https://www.R-project.org/
